# Accumulation characteristics of mineral elements in the fruiting bodies of *Lentinula edodes* from main production areas in China

**DOI:** 10.3389/fnut.2026.1851650

**Published:** 2026-06-16

**Authors:** Dandan Zhai, Tao Yuan, Meina He, Yang Fu, Ning Jiang, Tengye Luan, Meiyan Zhang, Guirong Tang, Xiaodong Shang, Yu Li, Xiaoxia Song, Hailong Yu

**Affiliations:** 1 Institute of Edible Fungi, Shanghai Academy of Agricultural Sciences, National Engineering Research Center of Edible Fungi, Shanghai, China; 2 Engineering Research Centre of Chinese Ministry of Education for Edible and Medicinal Fungi, Jilin Agricultural University, Changchun, China; 3 Institute of Agricultural Science and Technology Information, Shanghai Academy of Agricultural Sciences, Shanghai, China

**Keywords:** bioaccumulation, dietary source, essential minerals, food safety, *Lentinula edodes*

## Abstract

**Introduction:**

China is the world’s leading producer of *Lentinula edodes*, the most widely consumed edible mushroom and an important dietary element source. However, no nationwide study has systematically assessed its mineral profile, including content ranking, elemental enrichment/absorption patterns, and the respective influences of origin, cultivar, and heavy metals.

**Methods:**

Ten essential minerals (K, Ca, Na, P, Mg, Cu, Fe, Se, Mn, Zn) were analyzed in *L. edodes* fruiting bodies. Samples were collected from 44 production bases across 39 major production regions in China, all cultivated under a standardized substrate formula.

**Results:**

The average mineral contents ranked as follows: K > P > Mg > Ca > Na > Zn > Fe > Mn > Cu > Se. K, P, and Se were enriched from substrate, whereas Ca, Mg, Na, Fe, and Mn were absorbed. Origin influenced K, Mg, P, and Cu; cultivar affected K, P, and Cu; heavy metals impacted Ca, Fe, Zn, and Se. Notably, K, Mg, P, Mn, Cu, and Zn contributed meaningfully to dietary recommendations for the age groups of 7, 18, and 75 years.

**Discussion:**

*L. edodes* exhibits a favorable high-K, low-Na dietary profile, and its high Se enrichment activity makes it a promising candidate for Se-enriched mushroom products.

## 1 Introduction

Mineral elements are essential inorganic nutrients for living organisms, classified into macronutrients (e.g., C, H, O, N, P, K, Ca, Mg, S, and Na) and micronutrients (e.g., Fe, Cu, Mn, Zn, Se, Co, Mo, Cr, Ni, F, and I) based on their contents and physiological demand in the human body (1). These mineral elements directly participate in and regulate a variety of fundamental biological processes, including but not limited to: energy metabolism (e.g., ATP synthesis and electron transfer mediated by Mg and P), tissue structure construction (e.g., skeletal mineralization and cell membrane integrity involving Ca and P), redox homeostasis (e.g., antioxidant enzyme systems dependent on Se, Cu, and Mn), immune defense (e.g., immune cell function regulated by Zn and Se), and neural signal transduction (e.g., membrane potential and synaptic transmission maintained by K, Na, and Mg) (2–7). It is noteworthy that these essential mineral elements cannot be synthesized by the human body itself and must be obtained through dietary intake to meet physiological needs. Studies have shown that mineral deficiencies can lead to specific diseases, such as Fe-deficiency anemia, Ca-vitamin D-deficiency rickets, and Se-deficiency Keshan disease, and may even pose life-threatening risks (8–12). However, indiscriminate supplementation with mineral preparations may result in excessive intake, which not only fails to confer the expected health benefits but may also adversely affect immune homeostasis (13, 14). Therefore, diversified dietary intake, especially the consumption of foods rich in high-quality proteins and bioavailable minerals, is the ideal nutritional strategy (15, 16). Compared with green vegetables, edible mushrooms contain higher levels of essential mineral elements for humans and represent a superior dietary source of minerals (17, 18).

Different mushroom species exhibit significant differences in mineral element content and demonstrate distinct absorption preferences for various elements (19, 20). *Lentinula edodes* (shiitake mushroom) is the most widely cultivated and consumed edible mushroom globally, valued not only for its flavor but also for its bioactive compounds with anti-tumor and hypoglycemic properties (21–23). Compared with other edible fungi, *L. edodes* offers a more balanced mineral nutritional profile (19). China is the dominant producer, accounting for over 95% of the global supply since 2018 (24), with cultivation practices varying significantly in climatic conditions, substrate formulas, cultivars, and techniques. Previous studies have investigated the mineral content of *L. edodes* fruiting bodies in relation to regions, cultivars, substrate formulations, and flushes (25–28). However, these studies have been limited in geographic scope and have not systematically examined the relationship between mineral content in fruiting bodies and their substrates across major production areas under standardized conditions. Consequently, the key factors driving the variation in mineral content of Chinese *L. edodes* remain poorly understood, hindering efforts to promote it as a reliable dietary source of minerals.

To address this knowledge gap, the present study investigated the accumulation characteristics of 10 essential mineral elements (K, Ca, Na, P, Mg, Cu, Fe, Se, Mn, Zn) in *L. edodes* fruiting bodies collected from 44 production bases across 39 major production counties in China. The objectives were to determine the concentrations of these elements, evaluate their nutritional contribution based on Chinese Dietary Reference Intakes, analyze the transfer patterns from substrate to fruiting bodies, and assess the influence of origin, cultivar, and heavy metals on mineral content variability.

## 2 Materials and methods

### 2.1 Sample sources

**Figure 1:**
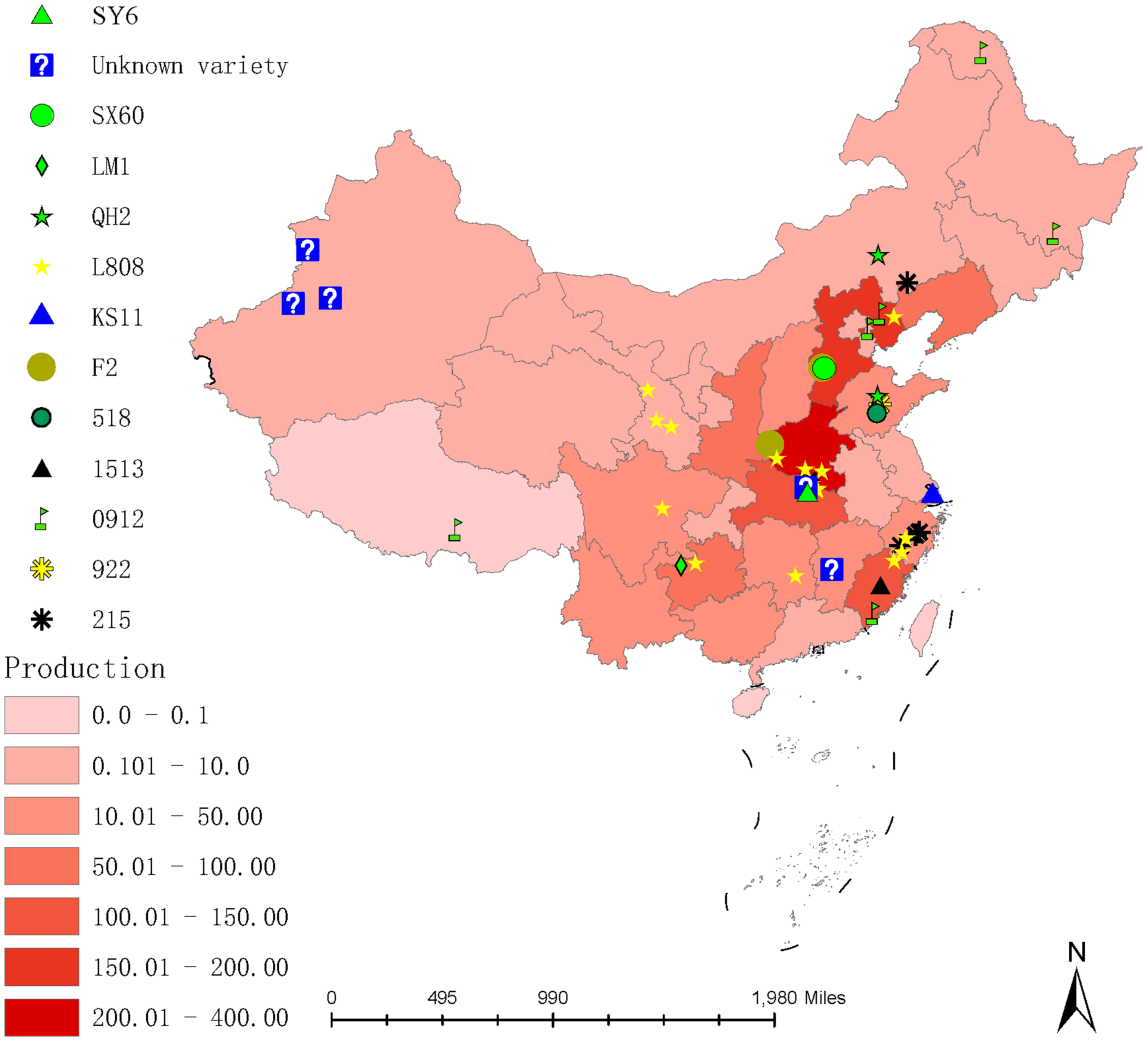
Sampling sites and corresponding cultivar distribution of the 44 *L. edodes* samples. Different markers represent different sampling sites and their corresponding cultivars. *L. edodes* production data were obtained from the China Edible Fungi Association (unit: 10^4^ tons). Adapted from standard base map GS (2024) 0650, downloaded from the Tianditu official website (https://www.tianditu.gov.cn/). The map file was converted using the mapshaper website (https://mapshaper.org/) to ensure compatibility, and the yield data and sampling site locations were overlaid using ArcGIS 10.8.1 software. No modifications were made to the national boundaries, provincial boundaries, or any other base map content.

To minimize variability in cultivation substrates, a unified substrate formulation (79% hardwood sawdust, 20% wheat bran, and 1% gypsum) was established as the screening criterion. Based on industrial production data from 2019 to 2023 provided by the China Edible Fungi Association, production bases adopting this specific formulation were identified nationwide. According to production yields (with higher-yielding provinces allocated more sampling sites), a stepwise screening process was conducted, ultimately delimiting the sampling area to 39 major production counties across 15 provinces and 2 municipalities in China (Henan, Hebei, Zhejiang, Hubei, Hunan, Fujian, Jiangxi, Shandong, Sichuan, Gansu, Heilongjiang, Inner Mongolia, Tibet, Xinjiang, Guizhou, Shanghai, and Tianjin). Within each selected county, eligible production bases were randomly chosen for the collection of *L. edodes* fruiting bodies and their corresponding cultivation substrates, resulting in a total of 44 sample sites. The 44 samples comprised 13 cultivars: L808 (*n* = 16), 0912 (*n* = 7), SX215 (*n* = 5), QH2 (*n* = 2), F2 (*n* = 2), SY6 (*n* = 1), SX60 (*n* = 1), LM1 (*n* = 1), KS11 (*n* = 1), 922 (*n* = 1), 518 (*n* = 1), 1,513 (*n* = 1), and unknown varieties (*n* = 5). Sampling locations and cultivar distribution are shown in Figure 1. The base map of China used in Figure 1 is from the Standard Map Service of the Ministry of Natural Resources of China (http://bzdt.ch.mnr.gov.cn). This sampling strategy allowed for the inclusion of samples from a range of origins, climatic conditions, cultivation models, and cultivars.

### 2.2 Sample processing

Fresh fruiting bodies and substrate samples were processed as described previously (29). Briefly, samples were cleaned with deionized water to remove surface contaminants, dried at 60 °C to constant weight, ground into a fine powder using a stainless steel grinder. The powdered samples were stored in sealed polyethylene bags in a desiccator at room temperature until analysis.

### 2.3 Mineral content measurement

Mineral element concentrations were determined according to Chinese national standards. K, Ca, Na, P, Mg, Cu, Fe, Zn, and Mn were analyzed by inductively coupled plasma mass spectrometry (ICP-MS) following GB 5009.268–2016. Se was determined by hydride generation atomic fluorescence spectrometry (HG-AFS) according to GB 5009.93–2017. For heavy metals, Pb and Cd were analyzed by graphite furnace atomic absorption spectrometry (GFAAS) following GB 5009.12–2017 and GB 5009.15–2014, respectively. Total Hg and As were determined by atomic fluorescence spectrometry (AFS) according to GB 5009.17–2014 and GB 5009.11–2014.

### 2.4 Nutritional assessment

The Index of Nutritional Quality (INQ) was used to evaluate whether *L. edodes* meets human nutritional requirements for minerals (30). The INQ was calculated as: INQ = (mineral content per 100 g/daily mineral requirement)/(energy content per 100 g/daily energy requirement). An INQ > 1 indicates that the food can satisfy dietary needs for that nutrient relative to its energy content, while an INQ < 1 indicates insufficiency. The average energy value of dried *L. edodes* was 1,433 kJ per 100 g (data source: China Food Composition Database, https://nlc.chinanutri.cn/fq/). Daily energy requirements and recommended mineral intakes for individuals aged 7, 18, and 75 years were obtained from the Chinese Dietary Reference Intakes (2023 edition). These age groups were selected to represent children, adults, and the elderly, respectively. For the INQ calculation, the daily energy requirements were taken as the mean values of males and females at the moderate physical activity level for each age group. The recommended mineral intakes for each age group were also taken as the mean values of males and females.

### 2.5 Statistical analysis

Data were processed using Excel. Spearman correlation analysis was conducted to examine relationships among mineral elements. The coefficient of variation (CV) was calculated as (standard deviation/mean) × 100%. Statistical significance was set at *p* < 0.05, *p* < 0.01, and *p* < 0.001. Figures were generated using Origin 2021.

## 3 Results

### 3.1 Mineral element contents and nutritional evaluation

**Figure 2:**
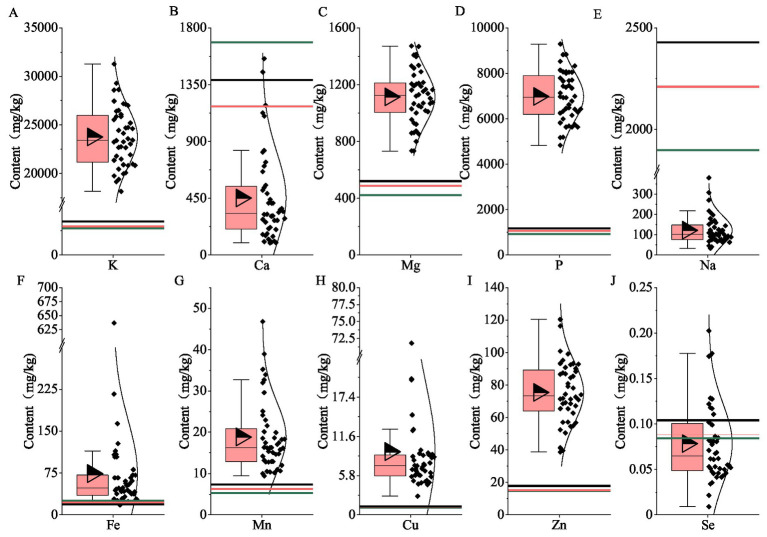
Content of 10 mineral elements in *L. edodes* fruiting bodies and different thresholds for meeting nutrient requirements of different age groups. Panels **A–J** represent K, Ca, Mg, P, Na, Fe, Mn, Cu, Zn, and Se, respectively. The horizontal lines represent the minimum content thresholds required to meet the recommended daily nutrient intake levels for different age groups. Green line: 7-year-old children; red line: 18-year-old adults; black line: 75-year-old elderly individuals.

The concentrations of 10 mineral elements in *L. edodes* fruiting bodies showed wide variation (Supplementary Table S1 and Figure 2). The mean contents, ranked from highest to lowest, followed the order: K (23,761 mg/kg) > P (6,976 mg/kg) > Mg (1,113 mg/kg) > Ca (452 mg/kg) > Na (122 mg/kg) > Zn (75.4 mg/kg) > Fe (73.6 mg/kg) > Mn (18.8 mg/kg) > Cu (9.34 mg/kg) > Se (0.079 mg/kg). K was the most abundant element, ranging from 18,145 to 31,270 mg/kg, while Se showed the lowest levels, ranging from 0.009 to 0.203 mg/kg.

Based on the 2023 Chinese Dietary Reference Intakes, the contents of K, Mg, P, Mn, Cu, and Zn in all 44 samples exceeded the recommended nutrient intakes for individuals aged 7, 18, and 75 years (Figure 2). In contrast, Na content in all samples fell below the recommended intake levels across all age groups, indicating that *L. edodes* alone cannot meet daily Na requirements. For Ca, no sample met the requirement for 7-year-old children, four samples (9.1%) met the requirement for 18-year-old adults, and two samples (4.5%) met the requirement for 75-year-old elderly individuals. For Fe, 39 samples (88.6%) met the requirement for 7-year-old children, while 43 samples (97.7%) met the requirements for both 18-year-old adults and 75-year-old elderly individuals. For Se, 14 samples (31.8%) met the requirement for 7-year-old children, 12 samples (27.3%) met the requirement for 18-year-old adults, and 10 samples (22.7%) met the requirement for 75-year-old elderly individuals.

### 3.2 Correlation analysis of mineral elements in fruiting bodies

**Figure 3:**
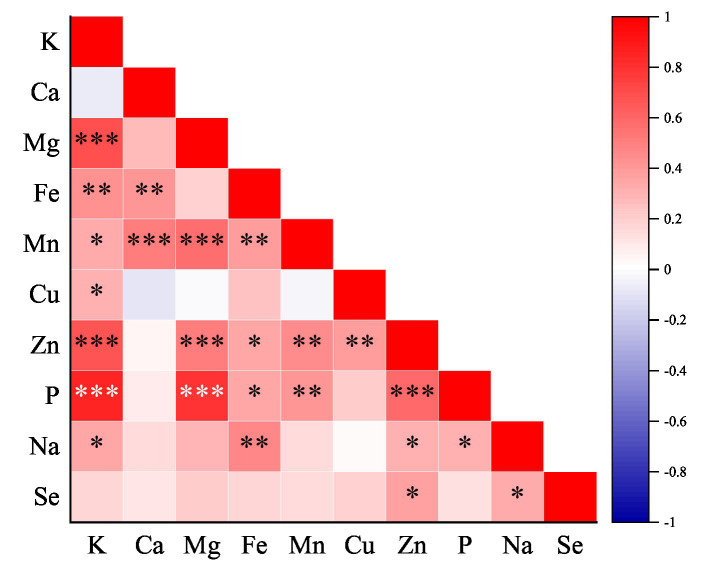
Correlation analysis of 10 mineral elements in *L. edodes* fruiting bodies. * denotes *p* < 0.05, ** denotes *p* < 0.01, and *** denotes *p* < 0.001.

To further clarify the relationships among the 10 mineral elements in *L. edodes* fruiting bodies, Spearman correlation analysis was performed. The results revealed significant positive correlations among all elements (Figure 3). Each element exhibited a distinct set of positive correlations with others. Zn showed positive correlations with eight elements (K, Mg, Fe, Mn, Cu, P, Na, Se); K with seven elements (Mg, Fe, Mn, Cu, Zn, P, Na); P, Mn, and Fe each with six elements (P with K, Mg, Fe, Mn, Zn, Na; Mn with K, Ca, Mg, Fe, Zn, P; Fe with K, Ca, Mn, Zn, P, Na); Na with five elements (K, Fe, Zn, P, Se); Mg with four elements (P, Zn, Mn, K); and Ca, Cu, and Se each with two elements (Ca with Fe, Mn; Cu with K, Zn; Se with Zn, Na). Notably, eight element pairs showed extremely significant correlations (*p* < 0.001): K-Mg, K-Zn, K-P, Mn-Ca, Mn-Mg, Mn-Zn, Mn-P, and P-Zn.

### 3.3 Transfer patterns of mineral elements from substrate to fruiting bodies

**Figure 4:**
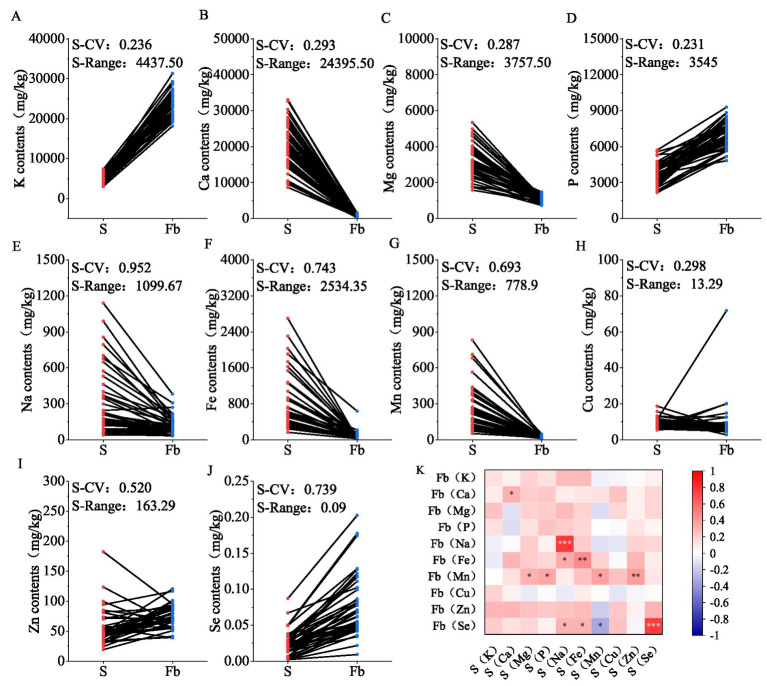
The effect of substrate on the mineral element content in fruiting bodies. Panels **A–J** represent K, Ca, Mg, P, Na, Fe, Mn, Cu, Zn, and Se, respectively, showing paired concentrations in substrate (S) and fruiting bodies (Fb). Panel K presents a heatmap of correlation coefficients between elements in substrate (S) and fruiting bodies (Fb). S: Substrate; FB: Fruiting body; S-CV: Coefficient of variation in substrate; S-Range: Range in substrate. * denotes *p* < 0.05, ** denotes *p* < 0.01, and *** denotes *p* < 0.001.

The substrate serves as the primary source of mineral elements for *L. edodes* fruiting bodies. To assess the absorption and accumulation patterns of the 10 elements, their contents were compared between paired substrate and fruiting body samples (Figures 4A–J). K, P, and Se exhibited significantly higher contents in fruiting bodies than in their corresponding substrates (Figures 4A,D,J), indicating active enrichment of these elements during growth; Ca, Mg, Na, Fe, and Mn showed lower contents in fruiting bodies than in substrates (Figures 4B,C,E–G), suggesting absorption without enrichment; Cu and Zn displayed inconsistent patterns across samples, with some showing enrichment and others showing depletion (Figures 4H,I), suggesting that their accumulation may be influenced by additional factors.

Further correlation analysis (Figure 4K) revealed element-specific relationships between fruiting body and substrate contents. No significant correlations were observed for K, P, Mg, Cu, or Zn. In contrast, significant positive correlations were found for Se (*r* = 0.757), Na (*r* = 0.772), Ca (*r* = 0.302), Mn (*r* = 0.363), and Fe (*r* = 0.441). Additionally, cross-element correlations were observed: Fe content in fruiting bodies was positively correlated with Na (*r* = 0.337) and Fe (*r* = 0.441) in substrate; Mn content in fruiting bodies showed positive correlations with substrate Mg (*r* = 0.307), P (*r* = 0.354), Mn (*r* = 0.363), and Zn (*r* = 0.390); and Se content in fruiting bodies was positively correlated with substrate Se (*r* = 0.757), Na (*r* = 0.306), and Fe (*r* = 0.311), but negatively correlated with substrate Mn (*r* = −0.326).

### 3.4 Influence of origin

**Figure 5:**
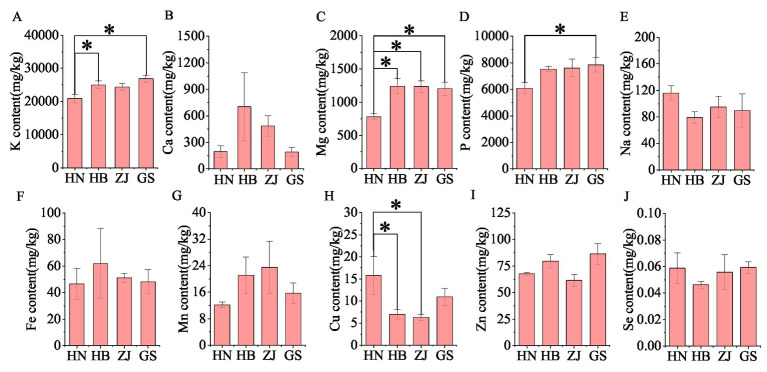
Variation in accumulated content of 10 mineral elements in *L. edodes* fruiting bodies across different origins. Panels **A–J** represent K, Ca, Mg, P, Na, Fe, Mn, Cu, Zn, and Se, respectively. HN, Henan province; HB, Hubei province; ZJ, Zhejiang province; GS, Gansu province. * denotes *p* < 0.05.

To assess origin effects, the widely cultivated L808 cultivar was compared across four provinces (Henan, Hubei, Zhejiang, and Gansu). Significant differences among origins were observed for K, Mg, P, and Cu (Figure 5). K content was significantly higher in samples from Hubei and Gansu than in those from Henan. Mg was significantly lower in Henan samples compared with the other three provinces. P was significantly higher in Gansu than in Henan. Cu was significantly higher in Henan than in Hubei and Zhejiang. No significant differences among origins were found for Ca, Na, Fe, Mn, Zn, or Se.

### 3.5 Influence of cultivar

**Figure 6:**
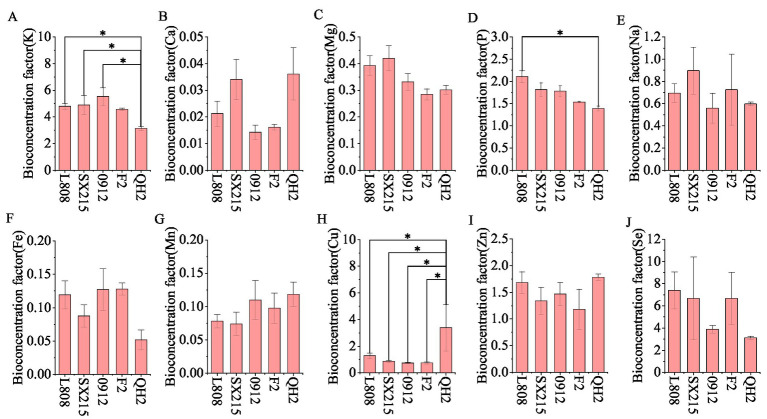
Variation in accumulated content of 10 mineral elements in *L. edodes* fruiting bodies across different cultivars. Panels **A–J** represent K, Ca, Mg, P, Na, Fe, Mn, Cu, Zn, and Se, respectively. *denotes *p* < 0.05.

Comparison of five widely cultivated cultivars (L808, SX215, 0912, F2, QH2) showed significant cultivar effects on K, P, and Cu accumulation (Figure 6). K enrichment capacity was significantly higher in L808, SX215, and 0912 than in QH2. P enrichment was significantly higher in L808 than in QH2. Cu accumulation was significantly higher in QH2 than in L808, SX215, 0912, and F2. No significant cultivar differences were observed for Se, Na, Zn, Mg, Fe, Mn, or Ca.

### 3.6 Influence of heavy metals

**Figure 7:**
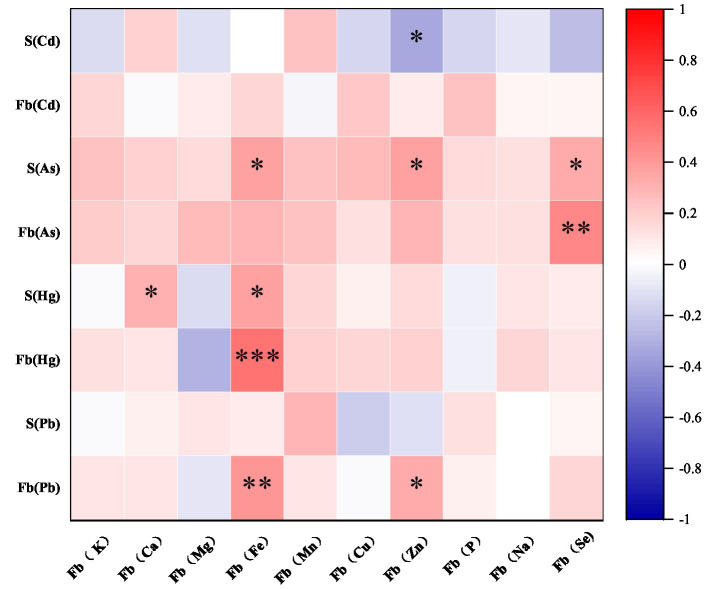
Correlation analysis between mineral elements in fruiting bodies and heavy metals in substrate and fruiting bodies. S, substrate; FB, fruiting body. * denotes *p* < 0.05, ** denotes *p* < 0.01, and *** denotes *p* < 0.001. Weak but statistically significant correlations (*r* < 0.2) are shown in the figure but are not discussed in detail due to limited biological relevance.

Correlation analysis between essential minerals in fruiting bodies and heavy metals in both substrates and fruiting bodies revealed that Ca, Fe, Zn, and Se showed correlations with certain heavy metals, whereas K, Mg, Mn, Cu, P, and Na exhibited no significant correlations with any heavy metal (Figure 7). Specifically, Ca content in fruiting bodies was positively correlated with Hg content in the substrate (*r* = 0.307). Fe content showed positive correlations with As in the substrate (*r* = 0.274). Zn content exhibited a negative correlation with Cd in the substrate (*r* = −0.237). Se content was positively correlated with As in both fruiting bodies (*r* = 0.478) and the substrate (*r* = 0.327). In addition to these notable correlations, several weak but statistically significant correlations (*p* < 0.05) were observed. However, due to their low correlation coefficients (*r* < 0.2), these are unlikely to represent biologically meaningful relationships and were not considered further.

## 4 Discussion

With the growing global consumption of *L. edodes*, attention to its mineral nutritional contents has increased. Li et al. (26) analyzed nine mineral elements in six cultivars under uniform cultivation conditions. However, their conclusions may not fully reflect the complex reality of China, the world’s leading producer. Given China’s long cultivation history, characterized by diverse strains, substrate materials, and production regions, localized or conditions restricted studies risk yielding partial or misleading conclusions. Therefore, acquiring broad spectrum data across major production areas is essential for reliably assessing mineral content and its influencing factors. To address this challenge, we adopted a widely accepted substrate formula and systematically collected samples from China’s main production regions, revealing for the first time the actual variation in mineral content from a multifactorial perspective encompassing origin, substrate, cultivar, and heavy metals. Nevertheless, we acknowledge two limitations. First, the sample sizes across different cultivars are imbalanced (e.g., L808, *n* = 16; several other cultivars, *n* = 1). While this reflects the actual production structure of China’s *L. edodes* industry, it may introduce statistical bias. Second, despite our efforts to standardize the substrate formulation, differences in raw material sources (e.g., sawdust tree species, wheat bran origin), local water quality, and environmental conditions across production regions may still influence the results. In fact, these objective regional factors, which are difficult to fully control, have imparted a strong regional signature to our data (Figure 5). This finding itself highlights the profound influence of production environment on mineral accumulation in *L. edodes*.

In this study, INQ was calculated using the mean energy and mineral requirements of males and females at the moderate physical activity level for three age groups (7, 18, and 75 years), representing children, adults, and the elderly, respectively. The moderate activity level represents the majority of the general population, and the use of sex-averaged values reflects a general nutritional evaluation of *L. edodes*. Notably, energy and mineral requirements vary across populations. Individuals with higher activity levels have increased energy demands, which may slightly lower the INQ values for Ca, Fe, and Se, while those with lower activity levels may see slightly higher values. However, for elements such as K, Mg, P, Mn, Cu, and Zn, INQ values exceed 1 across different sex, age, and activity level assumptions, indicating that the core conclusion is robust. For borderline elements (Ca, Fe, Se), interpretation should consider specific population characteristics. This study did not assess the bioavailability of mineral elements or analyze the potential effects of anti-nutritional factors on mineral absorption; therefore, the dietary recommendations are based solely on total mineral content. The primary objective of this study was to determine the actual mineral content in *L. edodes* from China’s main production areas and to identify its key influencing factors. Future studies will further evaluate mineral bioavailability in biofortification trials. Additionally, although mineral contents are reported on a dry weight basis, literature data consistently indicate that the moisture content of fresh *L. edodes* is approximately 90% (31), allowing reasonable conversion to fresh weight equivalents for dietary intake estimation.

**Figure 8:**
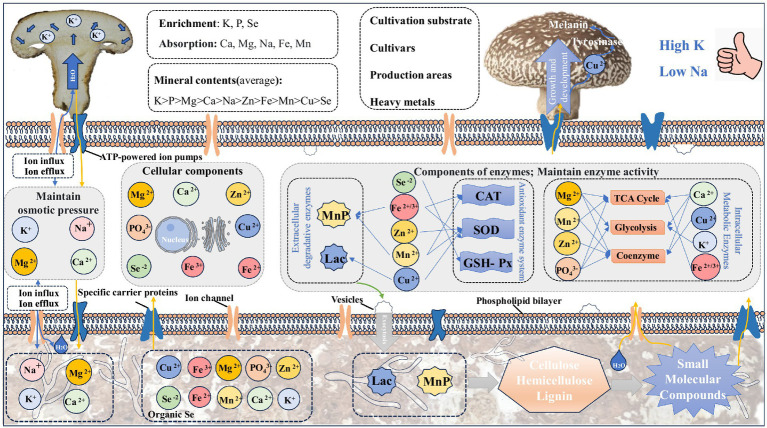
Schematic illustration of mineral element uptake, enrichment, and metabolic mechanisms in *L. edodes* fruiting bodies. Mnp, manganese peroxidase; Lac: laccase; CAT, catalase; SOD, superoxide dismutase; GSH-Px, glutathione peroxidase. Arrows indicate the direction of material flow, and black straight lines point to the names of corresponding cell structures.

The variation patterns of the nine mineral element contents in *L. edodes* observed in this study are consistent with those reported by Li et al. (26). Among the macroelements, K was the most abundant, followed by P, Mg, Na, Ca; among the microelements, Zn was the most abundant, followed by Fe, Mn and Cu (Figures 2, 8). Our INQ analysis further reveals that *L. edodes* is not only an excellent dietary source of beneficial minerals including K, Mg, P, Mn, Cu, and Zn, but also contains remarkably low levels of Na, with concentrations well below the recommended daily intake threshold across all age groups (Figures 2, 8). This distinctive high K and low Na nutritional profile positions *L. edodes* as a particularly healthy food option, benefiting cardiovascular and bone health (32–34). However, patients with hyperkalemia who cannot effectively metabolize K should monitor their intake of K rich foods (35, 36). Compared with mineral contents in other vegetables, edible fungi, particularly *L. edodes*, can serve as a healthy food option for individuals without underlying conditions such as hyperkalemia. Moreover, consumption of common mushrooms may reduce the risk of cancer and the likelihood of depression (37, 38).

Beyond these nine elements, Se, an essential mineral element in human growth, was also measured in this study. A Se-rich diet can alleviate cardiopulmonary oxidative stress, inflammation, and heart failure induced by excessive stress load (39). Fungal fruiting bodies can incorporate Se into proteins, polysaccharides, and nucleic acids, converting inorganic Se into organic forms (40). Leveraging the Se-enriching properties of edible fungi, Se-enriched products such as *Pleurotus ostreatus*, *Antrodia cinnamomea*, and *Auricularia auricula* have been developed to help meet the Se intake needs of Chinese residents through dietary consumption (41–43). This practice has been implemented in many regions across China (44–46). Although the Se content in *L. edodes* fruiting bodies was lower than that of the other nine elements, it exhibits a strong capacity for Se enrichment from substrate (Figure 4J). Therefore, supplementing Se in the cultivation substrate represents a promising strategy to produce Se enriched *L. edodes* fruiting bodies with enhanced nutritional value.

Mineral elements and organic nutrients are often mechanistically coupled in their transmembrane transport. The pathway of organic nutrients is relatively clear: the mycelium secretes extracellular enzymes to degrade the substrate, absorbs the released nutrients, then translocates them to the fruiting body for tissue formation (47–49) (Figure 8). Many of extracellular degrading enzymes contain mineral elements as cofactors, such as Mn-containing manganese peroxidase and Cu-containing laccase (50–52). Absorbed mineral elements serve as structural components of mycelial cells (e.g., P, Mg, Ca, Zn, Cu, Fe, Se) and participate in energy metabolism (e.g., P, Fe, Cu, Mg, Mn, Zn, K, Ca) (53). Specifically, K acts as an enzyme cofactor, P as a key component of nucleic acids and ATP, and Mg as an essential activator of glycolysis and the TCA cycle (53–55). Mineral elements are actively transported across hyphal cells via ion channels, carrier proteins, and ATP-powered pumps, and together with water, are translocated through the stipe to the cap, facilitating stipe elongation and cap expansion while accumulating in the fruiting body (46–48). The fruiting body, containing approximately 90% water, absorbs large amounts of mineral elements to maintain osmotic pressure, with K, Ca, Na, and Mg playing roles (31, 53). K, being the most abundant, is speculated to be the main osmotic regulator in *L. edodes* (56–58).

When cells are exposed to stress, they produce large amounts of reactive oxygen species (ROS). Excessively high concentrations of ROS can be strongly toxic to cells and may even lead to cell death (59). *L. edodes* encounters various abiotic stresses and has evolved a comprehensive antioxidant system, with mineral elements such as Fe, Mn, Se, and Zn serving as essential cofactors for enzymes like catalase (Fe and Mg), superoxide dismutase (Cu/Zn, Mn, or Fe), and glutathione peroxidase (Se) (60–64). Previous studies have shown that enzyme systems are involved in metal homeostasis in fungi, and based on the element-specific accumulation patterns observed here, we hypothesize that they may differentially respond to different mineral elements-a hypothesis requires further experimental validation. Additionally, when absorbing beneficial minerals, *L. edodes* also accumulates heavy metals, such as Cd and As (29, 65, 66). In plants, minerals alleviate Cd toxicity by competing for the same transporter channels, and Zn, Fe, Cu, and K are key minerals influencing Cd accumulation (67–69). In *L. edodes*, exogenous Ca supplementation has been found to reduce Cd content in the mycelium (70). This may be because Ca, which is required in large amounts, regulates energy metabolism (TCA cycle and glycolysis) and acts as a second messenger in fungal signal transduction (71, 72). Exogenous Ca likely inhibits Cd uptake through ion competition, and promote the efflux of Cd via calcium signal-regulated transport proteins, thereby reducing Cd levels in the mycelium.

## 5 Conclusion

Edible fungi contain higher levels of essential mineral elements for human health than vegetables, making them a superior source of dietary minerals. In this study, fruiting bodies of *L. edodes* and their corresponding substrates were collected from 39 major fungi-producing counties across 15 provinces and 2 municipalities in China, covering most of the country’s key production regions. This study investigated the accumulation characteristics of 10 mineral elements (K, Ca, Na, P, Mg, Cu, Fe, Se, Mn, Zn) in the fruiting bodies of *L. edodes*. In addition, the effects of key production factors under actual cultivation conditions (substrate, cultivars, origins, heavy metals) on mineral accumulation were evaluated. Results showed that the average mineral content in *L. edodes* fruiting bodies ranked as K > P > Mg > Ca > Na > Zn > Fe > Mn > Cu > Se. In terms of human nutritional needs, *L. edodes* fruiting bodies are a good dietary choice due to their high K and low Na content. The contents of K, Mg, P, Mn, Cu, and Zn are sufficient to meet the recommended intakes for individuals aged 7, 18, and 75 years. Most of the 10 mineral elements analyzed exhibited significant positive correlations in the fruiting bodies, suggesting coordinated accumulation among these elements under normal cultivation conditions. *L. edodes* demonstrated enrichment of K, P, and Se from the substrate, while it absorbed but did not enrich Ca, Mg, Na, Fe, and Mn. The contents of Se, Na, Ca, Mn, and Fe in the fruiting bodies were influenced by the composition of the substrate. Levels of K, Mg, P, and Cu in the fruiting bodies were significantly affected by origins. Cultivars significantly influenced the enrichment capacity of *L. edodes* for K, P, and Cu, indicating that mineral accumulation is also strongly affected by genetic factors. In practical cultivation environments, the levels of Ca, Fe, Zn, and Se in fruiting bodies were more susceptible to heavy metal influence, while the accumulation of K, Mg, Mn, Cu, P, and Na was less affected. This study not only analyzed the accumulation patterns of mineral elements in the fruiting bodies of *L. edodes* but also provides a scientific basis for selecting them as a high-quality dietary source of minerals.

## Data Availability

The original contributions presented in the study are included in the article/Supplementary material, further inquiries can be directed to the corresponding authors.
