# Supplementary material for: Accumulation characteristics of mineral elements in the fruiting bodies of Lentinula edodes from main production areas in China
Source: Front Nutr. 2026 Jun 16;13:1851650. doi: 10.3389/fnut.2026.1851650 (PMC13317020; doi:10.3389/fnut.2026.1851650)
Supplement: Supplementary file 1 [file Table_1.docx]

**Supplementary Data**

**Accumulation characteristics of mineral elements in the fruiting bodies of *Lentinula edodes* from main production areas in China**

**Table S1.** Concentration characteristics of 10 mineral elements in *L. edodes* fruiting bodies (dry weight)

| Mineral element | Concentration ranges（mg/kg) | Average value | Standard deviation | variation coefficient |
| --- | --- | --- | --- | --- |
| K | 18145.000-31270.000 | 23761.250 | 3056.501 | 0.129 |
| Ca | 96.400-1555.000 | 451.785 | 361.448 | 0.800 |
| Mg | 732.400-1472.000 | 1113.261 | 190.147 | 0.171 |
| Fe | 17.700-636.700 | 73.610 | 95.651 | 1.299 |
| Mn | 9.400-46.800 | 18.825 | 8.522 | 0.453 |
| Cu | 2.761-71.800 | 9.3393 | 10.260 | 1.099 |
| Zn | 38.700-120.400 | 75.412 | 18.843 | 0.250 |
| P | 4824.500-9282.000 | 6976.333 | 1097.346 | 0.157 |
| Na | 32.000-380.600 | 122.044 | 70.370 | 0.577 |
| Se | 0.009-0.203 | 0.079 | 0.044 | 0.558 |
